# Severe Myositis, Myocarditis, and Myasthenia Gravis with Elevated Anti-Striated Muscle Antibody following Single Dose of Ipilimumab-Nivolumab Therapy in a Patient with Metastatic Melanoma

**DOI:** 10.1155/2019/2539493

**Published:** 2019-04-30

**Authors:** Mahdieh Fazel, Patrick M. Jedlowski

**Affiliations:** University of Arizona College of Medicine – Tucson, 1501 N Campbell Ave, Tucson, AZ 85724, USA

## Abstract

Immune checkpoint inhibitors targeting programmed cell death protein 1 and cytotoxic T-lymphocyte associated protein 4 have improved survival in patients with metastatic melanoma, especially in combination (i.e., ipilimumab-nivolumab). Postmarketing surveillance has identified rare but at times life-threatening adverse effects associated with these agents in combination and as monotherapy, which include myocarditis, myositis, myasthenia gravis (MG), and hepatotoxicity. Further evaluation of immune checkpoint therapy-induced MG identified the rapid clinical progression, prolonged treatment/supportive therapy course, and higher frequency of myasthenic crisis in these patients versus those with idiopathic MG. More rapid incorporation of aggressive treatment options (i.e., intravenous immunoglobulin, plasmapheresis) may be necessary in these cases. Anti-striational antibodies are often detected in individuals with myasthenia gravis and concurrent myositis and myocarditis. A high-index of suspicion is necessary to assist with rapid treatment initiation as these patients can rapidly deteriorate into respiratory compromise. A case of a 78-year-old woman with metastatic melanoma status after combination therapy with ipilimumab-nivolumab that developed transaminitis, myositis, myocarditis, and myasthenia gravis (with positive anti-striational antibodies) five days after the first cycle, is presented. Despite high dose intravenous methylprednisolone and intravenous immunoglobulin treatment, she ultimately entered hospice care eight days after hospital admission, 36 days after her first cycle.

## 1. Introduction

Immune checkpoint inhibitors (ICIs) are a class of medications that include programmed cell death protein 1 (PD-1) inhibitors (nivolumab) and cytotoxic T-lymphocyte associated protein 4 (CTLA-4) inhibitors (ipilimumab) that disinhibit the immune system and antitumor immune response by blocking immune checkpoint cytokines [1]. The immune checkpoint molecules PD-1 and CTLA-4 have been found to be expressed on human cancers and serve to decrease T-cell activation and induce anergy [1]. The ICIs stimulate a robust immune response leading to a potent antineoplastic effect and several immune-related adverse effects (irAEs) including myositis, myocarditis, myasthenia gravis (MG), hepatotoxicity, hypothyroidism, and Miller-Fisher syndrome [1–3].

Myocarditis induced by ICIs, often occurring after the first or second cycle of therapy, has been reported in 1% of patients, with death occurring in half of the cases [4–6]. It cooccurs with myositis and MG in 25% and 11% of patients, respectively [4–6]. ICI-induced myocarditis and myositis can also be associated with concomitant MG, but overall neurologic irAEs occur in less than 1% of patients treated with ICIs [5, 7]. Here we report a rare case of nivolumab-ipilimumab induced MG (anti-striational antibody positive) with associated myositis, myocarditis, and transaminitis in a patient with metastatic melanoma.

## 2. Case Presentation

A 78-year-old woman with a past medical history significant for hypertension, intermittent asthma, prior pulmonary embolism, depression, and melanoma status after wide local excision four decades ago, was diagnosed with metastatic melanoma. Whole body positron emission tomography (PET) identified multiple metastatic lesions dispersed within the chest wall, lungs, lymph nodes, and axial skeleton. Combination immunotherapy with ipilimumab and nivolumab for four cycles, followed by nivolumab maintenance, was initiated.

Five days following the first cycle of combination immunotherapy, the patient developed diplopia and proximal muscle weakness/myalgias. Magnetic resonance imaging (MRI) was negative for metastatic disease within the brain or extraocular muscles. Given that her only other medications included amlodipine and escitalopram, it was hypothesized that these symptoms were adverse reactions to combination immunotherapy. Ipilimumab-nivolumab therapy was held and she received methylprednisolone intravenously (IV) in the clinic at a dose of 1 mg/kg body weight (75 mg).

Assessment in the hospital demonstrated abducens nerve, upward and downward gaze palsies, along with unsteady gait, and a diffuse rash. Patient had weakness and myalgias of proximal muscles bilaterally, greater in the lower extremities, and decreased vibratory sensation in the distal extremities. Vitamin B12 level was within normal limits and rapid plasma reagin (RPR) was nonreactive. Dosage of methylprednisolone was increased to 125 mg IV daily (1.5 mg/kg) due to severe clinical presentation. Routine dosing for acute myositis is methylprednisolone IV at 0.5-1.5 mg/kg; pulse therapy of 1000mg IV daily for 3 to 5 days in cases of severe myositis/lack of response or intravenous immunoglobulin (IVIG) can be initiated at 2 g/kg [8].

Labs demonstrated an elevated creatine phosphokinase (CPK) of 9198 IU/L, along with a transaminitis with an aspartate aminotransferase (AST) of 683 IU/L and an alanine aminotransferase (ALT) of 315 IU/L. C-reactive protein was elevated at 39.5 mg/L. Erythrocyte sedimentation rate and thyroid stimulating hormone (TSH) were within normal limits, and hepatitis panel was negative. Myositis panel was negative for myositis-related antibodies, including Jo-1, PL-7, PL-12, EJ, OJ, SRP, Mi-2 alpha, Mi-2 beta, MDA-5, TIF-1y, and NXP-2. Due to concern for immunotherapy-related myositis, methylprednisolone therapy was continued at a dose of 125 mg IV daily. Lower extremity MRI identified moderate edema of the subcutaneous tissue, superficial fascia, and muscles consistent with myositis. The patient's troponin-I level was 8.57 ng/mL. Transthoracic echocardiogram (TTE) was within normal limits, consistent with immunotherapy-related myocarditis.

The patient had persistent proximal muscle weakness and worsening gaze palsies that were minimally responsive to steroid therapy. The dose of methylprednisolone was increased from 125 mg IV to pulse steroid dosing of 1000 mg IV daily, for a total of three days. The patient developed new bulbar symptoms including dysphagia, voice hoarseness, and ultimate respiratory distress, and inability to manage secretions. Due to bulbar symptoms refractory to high dose steroid therapy, IVIG was started at a dose of 2 g/kg IV daily for two days. A summary of the patient's treatment during her hospital stay can be seen in Table 1.

Muscle weakness mildly improved with IVIG and IV steroid therapy, but bulbar symptoms progressively worsened. IVIG therapy for severe myositis is generally loaded at 2 g/kg IV for one to two doses and then repeated every four to eight weeks. Given that the patient had already received two loading doses of IVIG with worsening clinical status, plasmapheresis was initiated. Anti-GQ1B was tested to evaluate for Miller-Fisher syndrome and was negative. Patient developed bilateral ptosis and increased respiratory effort, necessitating transfer to the intensive care unit (ICU). Anti-striational antibodies were found to be positive, with an elevated titer of 1:80, supporting a diagnosis of ICI-induced MG.

The patient's clinical condition continued to deteriorate, with progressively worsening dysphagia, increased respiratory effort, decreased ability to manage respiratory secretions, complete gaze palsy, severe ptosis, and continued proximal muscle weakness. CPK, AST, ALT, and troponins were down-trending despite worsening symptoms (Table 2). Due to increased work of breathing, there was concern that the patient would soon require intubation. The patient expressed desire to cease aggressive therapy at that time and was evaluated by the palliative care team. She was deemed to have full decision making capacity and a prognosis of days to weeks. She elected to enter hospice care, and given her increased respiratory effort and inability to manage respiratory secretions, she was discharged to inpatient hospice care.

## 3. Discussion

ICI therapy has been efficacious in the treatment of many cancers including metastatic melanoma. Despite this success, ICI therapy is associated with multiple irAEs, some of which are rare but life-threatening including myocarditis, myositis, MG, and transaminitis as highlighted in this case [4]. MG occurs in 0.12% of patients treated with nivolumab monotherapy, but seldom with ipilimumab monotherapy [3]. In comparison to idiopathic MG, patients with nivolumab-induced MG (nivoMG) tend to have higher serum CPK levels and incidence of myocarditis, myositis, and MG [3]. Patients with nivoMG necessitated more aggressive therapy including IVIG and respiratory support, with a mean duration of 54 days [3]. In this case, methylprednisolone, IVIG, and plasmapheresis were ineffective in managing bulbar symptoms, consistent with documented cases of ICI-induced MG. Accordingly, earlier initiation of advanced treatment measures should be considered when suspecting ICI-induced MG.

Various etiologies could contribute to diplopia and proximal muscle weakness/myalgias. Therefore, a thorough clinical history and laboratory evaluation are required to delineate the etiology. A myositis panel could identify antibodies associated with polymyositis, dermatomyositis, and malignancy-associated myositis. An anti-GQ1B can be attained to evaluate for Miller-Fisher syndrome [2]. Anti-acetylcholine receptor antibodies can be identified in both ICI-induced and idiopathic MG [3]. In idiopathic MG, anti-striational antibodies are associated with myositis and/or myocarditis, suggesting that a similar pathogenic process may occur in irAEs [9]. More severe outcomes, including fatality, were seen in ICI-induced MG with anti-striational antibodies in comparison to seronegative cases [10].

This case demonstrates the importance of early screening for and management of irAEs associated with ipilimumab-nivolumab therapy as patients can develop rapidly progressive MG with subsequent myasthenic crisis. Additionally, clinicians should be aware of the more aggressive treatment and prolonged supportive care often required by patients with ICI-induced MG, especially those with positive anti-striational antibodies, and transition to IVIG/plasmapheresis earlier in the treatment plan. This case further highlights the possible role of anti-striational antibody detection in prognosticating patients with ICI-induced MG.

## Figures and Tables

**Table 1:** Treatment during hospital stay by day.

Treatment	1 (Admission)	2	3	4	5	6	7	8
Methylprednisolone IV	75 mg	125 mg	125 mg	1000 mg	1000 mg	1000 mg	150 mg	75 mg

IVIG					2 mg/kg	2 mg/kg		

Plasmapheresis							1^st^ cycle	

**Table 2:** Selecting laboratory values during hospital stay by day.

	1 (Admission)	2	3	4	5	6	7	8
AST	735	613	624	452	332	192	139	60

ALT	373	325	428	388	390	344	271	112

CK	9958	6995	5032	2858	1883	988	556	228

TSH	0.65							

CRP	39.5							

Troponin I				8.57 → 7.56	4.86	4.28	2.80	1.82

BNP					133			

ESR	9							

## Abbreviations

PDL-1: Programmed cell death protein 1
CTLA-4: Cytotoxic T-lymphocyte associated protein 4
MG: Myasthenia gravis
ICIs: Immune checkpoint inhibitors
irAEs: Immune-related adverse effects
PET: Positron emission tomography
MRI: Magnetic resonance imaging
IV: Intravenously
RPR: Rapid plasma reagin
CPK: Creatine phosphokinase
AST: Aspartate aminotransferase
ALT: Alanine aminotransferase
TSH: Thyroid stimulating hormone
TTE: Transthoracic echocardiogram
IVIG: Intravenous immunoglobulin
nivoMG: Nivolumab-induced MG.
